# Labor stimulation with oxytocin: effects on obstetrical and neonatal outcomes

**DOI:** 10.1590/1518-8345.0765.2744

**Published:** 2016-07-25

**Authors:** Pedro Hidalgo-Lopezosa, María Hidalgo-Maestre, María Aurora Rodríguez-Borrego

**Affiliations:** 1 PhD, Associate Professor, Facultad de Enfermería, Universidad de Córdoba, Córdoba, Spain. Instituto Maimónides de Investigación Biomédica de Córdoba (IMIBIC), Córdoboa, Spain.; 2 RN, Facultad de Enfermería, Universidad de Córdoba, Córdoba, Spain.; 3 PhD, Full Professor, Facultad de Enfermería, Universidad de Córdoba, Córdoba, Spain. Instituto Maimónides de Investigación Biomédica de Córdoba (IMIBIC), Córdoboa, Spain.

**Keywords:** Oxytocin, Labor, Newborn

## Abstract

**Objective:**

to evaluate the effects of labor stimulation with oxytocin on maternal and neonatal outcomes.

**Method:**

descriptive and analytical study with 338 women who gave birth at a tertiary hospital. Obstetric and neonatal variables were measured and compared in women submitted and non-submitted to stimulation with oxytocin. Statistics were performed using Chi-square test, Fisher exact test, Student t-test; and crude Odds Ratio with 95% confidence interval were calculated. A p < 0.05 was considered statistically significant.

**Results:**

stimulation with oxytocin increases the rates of cesarean sections, epidural anesthesia and intrapartum maternal fever in primiparous and multiparous women. It has also been associated with low pH values of umbilical cord blood and with a shorter duration of the first stage of labor in primiparous women. However, it did not affect the rates of 3rd and 4th degree perineal lacerations, episiotomies, advanced neonatal resuscitation, 5-minute Apgar scores and meconium.

**Conclusion:**

stimulation with oxytocin should not be used systematically, but only in specific cases. These findings provide further evidence to health professionals and midwives on the use of oxytocin during labor. Under normal conditions, women should be informed of the possible effects of labor stimulation with oxytocin.

## Introduction

Oxytocin is the most frequently medication used for labor induction in obstetrics^1^. Among the known benefits of its use is the improvement of contractions^2^. Oxytocin is commonly used in modern obstetric practice to increase uterine activity, in cases in which the labor process has failed, with the aim to enable it to progress to a vaginal delivery^3^.

The use of oxytocin has been indicated for the treatment of labor dystocia, as it may reduce the rates of cesarean sections^4^. Prolonged labor or dystocia has been described as one of the main indications for cesarean section, in situations in which there is cessation in the process that would result in a normal and spontaneous delivery^4^. 

In 2007, the Institute for the Safety of Medical Practice warned that oxytocin is a medicine that requires great caution^5^. This type of medication is characterized by requiring special attention and caution during its administration, since it presents a high risk of harm when incorrectly used. Errors related to the use of oxytocin are currently the most common that may occur during childbirth^5^. These errors are related to high doses in most cases, which may cause excessive uterine activity^6^. 

Interventions with oxytocin, particularly at high doses, may have potential adverse effects on the mother and the fetus, such as uterine tachysystole and impairment of fetal heart rates^3^. This occurs due to the reduction or interruption of the blood flow to the intervillous space during contractions^7^. Contractions in normal deliveries are well tolerated by the majority of fetuses; however, there is a risk of fetal hypoxemia and acidemia if the contractions are very frequent and/or prolonged^2^ ^,^ ^8^. Roman and Lothian^9^ concluded in their study that in the absence of complications, interventions during the physiological birth process increases the risk of changes for the mother and the fetus. They proposed the use of evidence-based clinical practice to promote physiological birth, avoiding unnecessary childbirth induction and use of regular care interventions, as well as unnecessary restrictions. However, a meta-analysis with 10 randomized controlled trials^10^ concluded that high doses of oxytocin for labor stimulation was associated with reduction in the rates of cesarean sections and shorter duration of labor, without increasing the maternal and perinatal adverse outcomes.

The World Health Organization stressed the need to revise the biomedical care model during pregnancy and childbirth, which is characterized by a high interventionism and excessive medicalization in developed countries. In its recommendations on the care during normal birth, certain practices and interventions were considered inadequate, such as amniotomy or early artificial rupture of the amniotic sac and regular use of oxytocin, among other^11^.

In Spain, in the Normal Birth Care Strategy, the Ministry of Health has recommended the limited use of oxytocin^12^.

Labor stimulation with oxytocin and early amniotomy used to be carried out regularly in the hospital where this study was conducted, a tertiary hospital in the South of Spain; however, their use have been currently reduced.

The aim of this study was to evaluate the effects of labor stimulation with oxytocin on the maternal and neonatal outcomes. The specific objectives were: to compare the rates of cesarean sections, 5-minute Apgar scores, arterial pH values of umbilical cord blood and type of neonatal resuscitation required, between women submitted and non-submitted to stimulation with oxytocin.

## Methods

This is a descriptive and analytical study carried out in a tertiary hospital in the South of Spain, with 338 women who gave birth from September 2011 to September 2013. This is a highly specialized hospital of regional and national reference, which primarily serves women from the city and downtown area of the province. About 4,000 women are assisted a year for childbirth in this hospital.

The study population was comprised of all women, primiparous and multiparous, with spontaneous labor onset assisted during this period. 

Inclusion criteria were: delivery at full term, low-risk pregnancy and childbirth, spontaneous onset of labor, single delivery with cephalic presentation. Women with induced labor and those who were not in labor due to elective or emergency cesarean section were excluded. 

Two groups were established, women whose labor was stimulated with oxytocin and women who did not receive oxytocin and whose delivery progressed spontaneously. Different obstetrical and neonatal variables were measured and compared between the two groups. 

Stimulation with oxytocin is defined as the administration of oxytocin to improve and/or increase the frequency and intensity of contractions in women whose delivery begins spontaneously. Oxytocin perfusion consisted of a dilution of five units of oxytocin in 500 ml of saline. The perfusion started with the use of 6 ml/h, which was doubled every 30 minutes up to a maximum of 96 ml/h, until achieving adequate contractions. It is used in women with spontaneous labor onset, in situations in which there is low frequency and/or intensity of uterine contractions or when the expansion process has failed and not progressed; although it is also used in other cases to increase uterine activity and thus accelerate the delivery process.

Participants were selected by systematic random sampling, by selecting a woman in every 25 birth registrations. The data of women who were selected for the sample were retrospectively obtained from medical records and treated anonymously. Data were entered into a database of the Statistics PASW program (version 18), with which the analysis was carried out.

The variables considered were: maternal age (years), parity (primiparous/multiparous), gestational age (weeks), antecedent of cesarean section (yes/no), 3^rd^ and 4^th^ degree vaginal-perineal lacerations (yes/no), epidural anesthesia (yes/no), use of oxytocin during the first stage of labor (yes/no), type of delivery (eutocic/instrumental/cesarean section), amniotomy (yes/no), intrapartum maternal fever (yes/no), advanced neonatal resuscitation (yes/no), 5-min Apgar test scores < or = 7 (yes/no), arterial pH values of umbilical cord blood < or = 7.20 (yes/no), presence of meconium in the amniotic fluid (yes/no) and duration of the first stage of labor (hours).

For data analysis, the statistical Chi-square and Fisher exact tests were used for qualitative variables, and Student's t-test was used for quantitative variables. The first are expressed as frequencies and percentages, whereas the latter are expressed as mean and standard deviation. Crude Odds Ratios with 95% Confidence Interval were also calculated for each variable. P<0.05 was considered statistically significant.

The Ethics Committee of the same hospital where this research was carried out approved the study design.

## Results

Of the 363 women selected for the study sample, 25 were excluded. Of these, 7 were excluded due to antecedents of cesarean sections, 6 due to emergency cesarean sections without labor, other 2 gave birth in a different hospital, other 7 due to premature birth, and finally, 3 due to delivery of twins. The final sample consisted of 338 women.

The average age of women in the sample was 30.80 (5.01) years, with a minimum age of 16 years and maximum of 46 years. The percentages of spontaneous births, instrumental births and cesarean sections were 67.5%, 13.9% and 18.6%, respectively. Primiparous women were 63% (n = 213) and multiparous were 37% (n = 125). In total, 6% of women (n = 20) had experienced a previous cesarean section. The percentage of women who were stimulated with oxytocin was 51.5% (n = 174). Likewise, the percentage of women receiving epidural anesthesia was 78% (n = 263). Episiotomy was performed in 39% of women (n = 133) and 11.5% (n = 39) had intrapartum fever. Early artificial rupture of membranes was performed in 48.5% of women (n = 164). The average of arterial pH of umbilical cord blood was 7.28 (0.09). These data are shown in Table 1. 

**Table 1:** Characteristics of women who gave birth in the study hospital (N = 338), Cordoba, CAA, Spain, 2011-2013

Variable		n (%)
Parity		
	Primiparous	213 (63)
	Multiparous	125 (37)
Type of delivery		
	Eutocic	228 (67.5)
	Instrumental	47 (13.9)
	Cesarean section	63 (18.6)
3^rd^ and 4^th^ degree lacerations		7 ( 2)
Prior cesarean section		20 (6)
Epidural anesthesia		263 (78)
Episiotomy		133 (39)
Use of oxytocin		174 (51.5)
Early amniotomy		164 (48.5)
Maternal intrapartum fever		39 (11.5)
Meconium		41 (12)
Umbilical cord pH mean (SD)^*^		7.28 (0.09)

* Data expressed as mean (Standard Deviation)

Regarding the obstetrical and neonatal outcomes (Table 2 shows the results of primiparous women, and table 3 shows the results of multiparous women). Statistically significant differences were found by comparing the results of the rates of cesarean section, epidural analgesia, intrapartum fever, arterial pH of umbilical cord blood and duration of the first stage of labor in primiparous women. There were significant differences in the use of epidural analgesia among multiparous women.

**Table 2:** Obstetric and neonatal results of primiparous women submitted and non-submitted to stimulation with oxytocin (N = 213). Cordoba, CAA, Spain, 2011-2013

Variable	Stimulation				
	SI	NO			
	n = 124	n = 89			
	n (%)	n (%)	p	crude OR	95%CI
Type of delivery					
Cesarean section	45 (36)	8 (9)	< 0.001	5.76	2.55-13.0
Instrumental	19 (15)	20 (22.5)	0.183	0.62	0.31-1.25
Episiotomy	50 (43)	47 (53)	0.094	0.60	0.34-1.04
3^rd^ and 4^th^ degree Lacerations	2 (1.5)	3(3.5)	0.652	0.47	0.07- 2.87
Maternal fever	26(21)	7 (8)	0.009	3.10	1.28-7.32
Epidural	115(93)	66(74)	< 0.001	4.45	1.94-10.19
Umbilical cord pH<=7.20	31 (25)	9 (11)	0.009	2.84	1.27-6.34
5-m Apgar < = 7	3 (2.5)	1 (1)	0.672	2.18	0.22-21.3
Advanced resuscitation	14 (10)	4 (4.5)	0.076	2.72	0.6-8.59
Meconium	21(22)	7 (11)	0.074	2.28	0.90-5.73
Duration 1^st^ stage labor^*^ (hours)	5.1(1.5)	6.8(2.6)	< 0.001	1.62	0.95-2.29

*Data expressed as mean (Standard Deviation)

**Table 3:** Obstetric and neonatal results of multiparous women submitted and non- submitted to stimulation with oxytocin (N = 125). Cordoba, CAA, Spain, 2011-2013

Variable	Stimulation				
	SI	NO			
	n = 124	n = 89			
	n (%)	n (%)	p	crude OR	95%CI
Type of delivery					
Cesarean section	8 (16)	2 (3)	0.014	6.95	1.41-34.27
Instrumental	5(10)	3 (4)	0.265	2.66	0.60-11.70
Episiotomy	18 (36)	18 (24)	0.147	1.78	0.81-3.90
3^rd^ and 4^th^ degree Lacerations	1 (2)	1 (1.3)	0.980	1.51	0.09-24.7
Maternal fever	5 (10)	1(1.5)	0.037	8.22	0.93-72.6
Epidural	46 (92)	36 (48)	< 0.001	12.45	4.07-38.0
Umbilical cord pH<=7.20	12 (25)	10 (13.5)	0.107	2.13	0.83-5.42
5-m Apgar < = 7	1 (2)	1 (1)	0.980	1.51	0.09-24.7
Advanced resuscitation	4 (9)	2 (3)	0.219	3.14	0.55-17.9
Meconium	6 (18)	7 (14)	0.608	1.36	0.41-4.49
Duration 1^st^ stage labor^*^ (hours)	4.1(1.4)	4.2(1.8)	0.821	0.08	-0.63-0.80

* Data expressed as mean (Standard Deviation).

Analysis of the type of delivery resulted in statistically significant differences among primiparous women, and it was verified a higher proportion of vaginal deliveries in the group of women non-submitted to stimulation with oxytocin. The percentage of cesarean sections among primiparous women non-submitted to stimulation was 9%, significantly lower than the percentage observed for those women stimulated with oxytocin (36%), resulting in significant differences (OR 5.76, 95%CI: 2.55-13, p <0.001). Similarly, multiparous women not stimulated with oxytocin showed a lower percentage of cesarean sections (OR 6.95, 95%CI: 1.41-34, p = 0.014).

Regarding the 3^rd^ and 4^th^ degree lacerations and episiotomies, no differences were observed between the two groups in primiparous and multiparous women. However, the percentage of primiparous women with epidural anesthesia in the group submitted to stimulation with oxytocin was 93% (n = 115) versus 74% (n = 66) in women non-submitted to stimulation with oxytocin (OR 4.45, 95%CI: 1.94-10.19, p <0.001). These differences were higher in multiparous women, 92%, when compared with 48% (OR = 12.45, 95%CI: 4.07-38, p <0.001). Significant differences were also found on the variable intrapartum fever. The percentage of women who had temperature of 38°C or higher during childbirth in the group submitted to stimulation with oxytocin was 21%, compared to 8% among women without oxytocin (OR 3.10, 95%CI: 1.28-7.52, p = 0.009). 

Regarding the neonatal results, significant differences were observed in the pH of umbilical cord blood in primiparous, since the percentage of newborns with pH <7.20 of mothers stimulated with oxytocin was 25%, compared with 11% in women who did not receive oxytocin (OR 2.84, 95%CI: 1.27-6.34, p = 0.009). No significant differences were observed among multiparous (OR 2.13, 95%CI: 0.83-5.42; p = 0.107). The average pH values were compared between the two groups, 7.26 (0.95) in the group submitted to stimulation with oxytocin versus 7.30 (0.93), p <0.001; and the mean difference was 0.036.

There were no significant differences in 5-min Apgar scores <= 7 among newborns of primiparous, 1% in the group submitted to stimulation compared with 2.5% (OR 2.18, 95% CI: 0.22 - 21, p = 0.672 ). Similarly, there were no significant differences among multiparous.

Regarding the variable advanced neonatal resuscitation, which requires endotracheal intubation, cardiac massage or administration of medications, or all of them, there were no significant differences in parity analysis. However, differences were observed when the whole sample was analyzed, primiparous and multiparous. In this regard, the rates of newborns who required such resuscitation in the group submitted to stimulation with oxytocin was 10.7% vs. 3.8% (OR = 3.0, 95% CI: 1.15 - 7.76, p = 0.018). No significant differences were found on the variable meconium in the amniotic fluid.

Finally, there were significant differences in the duration of the first stage of labor among primiparous. The average duration of this phase in the group submitted to stimulation with oxytocin in primiparous was 5.1 hours (1.5), compared with 6.8 hours (2.6) in the group non-submitted to stimulation with oxytocin (OR 1.62, 95% CI: 0,95 - 2.29, p <0.001). In multiparous women, these data were 4.1 h (1.4) compared to 4.2 (1.8), resulting in no significant differences. 

## Discussion

These results must be viewed with caution due to the limitations of this study. These include, that it was carried out in a single hospital, although it provides medical assistance to a large population. One might also consider that the sample is limited, which represents 8% of women treated in a year for delivery in this hospital. 

As previously mentioned, the objective of stimulation with oxytocin is to improve the uterine dynamics so that the labor progresses to a vaginal delivery^3^. However, the results obtained in this study indicate that in general, the use of oxytocin during labor may be related to adverse effects on the mother and the newborn. This is evidenced by the results regarding the rates of cesarean sections, epidural analgesia, intrapartum maternal fever, pH of the umbilical cord blood and advanced neonatal resuscitation, in primiparous. The perfusion of oxytocin for labor stimulation was significantly associated with an increase in the rates of cesarean sections, a higher percentage of intrapartum fever, lower pH of umbilical cord and greater need for advanced neonatal resuscitation. However, it was not associated with variations in the rates of instrumental delivery, 3^rd^ and 4^th^ degree lacerations and 5-min Apgar scores.

It seems coherent that the main differences have been found among primiparous women, because for them childbirth is usually longer, more difficult and may present more complications. In this context, some authors have reported that parity was a factor influencing the type of delivery and concluded that the normal or spontaneous labor is more common in multiparous than primiparous^13^.

In accordance with our obstetric results, other authors also concluded that the use of oxytocin does not reduce the rates of cesarean sections^1^, and others have concluded that it causes an increase^14^. In another study, the authors concluded that stimulation with oxytocin increased the chances of cesarean sections and instrumental deliveries using suction cup^15^. In contrast, other authors have shown an association between stimulation with oxytocin and decrease in the rates of cesarean sections^16^. In a meta-analysis with nine trials and 1,983 women, it was concluded that stimulation caused an increase in the number of vaginal deliveries^17^. In a 2011 review, the authors found no significant differences in the rates of cesarean sections or instrumental deliveries by using or not oxytocin^18^. Other authors also found no differences in their review, Cochrane^19^. On the other hand, in a recent study, the authors found that, using more traditional protocols with lower doses of oxytocin, it was observed a trend to an increase in the rates of cesarean sections^20^. 

The neonatal outcomes of this study are consistent with those of previous studies, in which statistically significant differences were observed when pH values of umbilical cord blood of newborns of mothers stimulated with oxytocin were compared with those of mothers non-submitted to stimulation. Accordingly, oxytocin use was associated with lower pH values^21^ ^-^ ^22^. In another study of cases and controls, carried out in 2013, the results of women with and without birth planning were compared and better results were observed in the pH of umbilical cord blood of newborns of mothers with birth planning, which were characterized by presenting a more spontaneous birth process and less use of oxytocin and other interventions^23^. Other studies have associated the use of protocols with high doses of oxytocin with higher rates of hospitalization in the neonatal unit, which is reduced when protocols with lower doses of oxytocin are used^20^.

Regarding the variable 5-min Apgar score, although in our study no significant differences were found, other authors found differences, namely a greater percentage of infants born vaginally from mothers stimulated with oxytocin, with Apgar scores per minute <= 7, compared with mothers non-submitted to stimulation^14^.

This study has also identified an association between stimulation with oxytocin and maternal intrapartum fever. However, other authors have not found enough evidence to sustain that high doses of oxytocin were related to maternal fever during labor^24^. In another study carried out in 2012^25^, it was found that high temperature was associated with a high body mass index and with the time elapsed from the rupture of the amniotic sac until delivery, whereas epidural analgesia did not influence on the temperature increase. In our study, no significant association between elevated maternal temperature and epidural administration was found.

The dilation stage proved to be longer in the group of primiparous women non-submitted to stimulation with oxytocin, so this result is consistent with those of other authors, which showed that the duration of this first stage of labor was significantly shorter when the dose of oxytocin was increased^1^.

## Conclusion

The results of this study show that the use of oxytocin in labor stimulation can be detrimental to both the mother and the newborn, since they indicate that the use of oxytocin is associated with increased cesarean section rates, use of epidural analgesia and maternal intrapartum fever, both in primiparous and multiparous. Furthermore, it was also observed a significant association between stimulation with oxytocin and low pH values of umbilical cord blood of newborns of primiparous mothers. On the other hand, coinciding with other studies, it has been proved its association with a shorter duration in the first stage of labor. However, it had no adverse effects on the rates of 3^rd^ and 4^th^ degree lacerations, episiotomies, advanced neonatal resuscitation, 5-min Apgar scores and meconium in the amniotic fluid.

Therefore, we may conclude that stimulation with oxytocin should not be used systematically, but only in very specific cases, in which its use is particularly necessary.

These results provide to health professionals a better understanding of the effects of the use of oxytocin during labor, which can be useful for decision-making in clinical practice. At the same time, these results reinforce the need to reflect on a change in the delivery care paradigm. In addition, they provide information to mothers towards the acquisition of more knowledge about the delivery process, considering that pregnant women should be informed of the possible effects of the use of oxytocin for labor stimulation.
